# Comparative Transcriptome and Endophytic Bacterial Community Analysis of *Morchella conica* SH

**DOI:** 10.3389/fmicb.2021.682356

**Published:** 2021-07-20

**Authors:** Bei B. Lü, Guo G. Wu, Yu Sun, Liang S. Zhang, Xiao Wu, Wei Jiang, Peng Li, Yan N. Huang, Jin B. Wang, Yong C. Zhao, Hua Liu, Li L. Song, Qin Mo, Ai H. Pan, Yan Yang, Xuan Q. Long, Wei D. Cui, Chao Zhang, Xu Wang, Xue M. Tang

**Affiliations:** ^1^Biotechnology Research Institute, Key Laboratory of Agricultural Genetics and Breeding, Shanghai Academy of Agricultural Sciences, Shanghai, China; ^2^Institute of Agriculture and Biotechnology, Zhejiang University, Hangzhou, China; ^3^Institute of Edible Fungi, Yunnan Academy of Agricultural Sciences, Yunnan, China; ^4^Institute of Edible Fungi, Shanghai Academy of Agricultural Sciences, Shanghai, China; ^5^Institute of Microbiology, Xinjiang Academy of Agricultural Sciences, Ürümqi, China; ^6^Translational Medical Center for Stem Cell Therapy and Institute for Regenerative Medicine, Shanghai East Hospital, School of Life Sciences and Technology, Tongji University, Shanghai, China; ^7^Department of Pathobiology, Auburn University, Auburn, AL, United States; ^8^HudsonAlpha Institute for Biotechnology, Huntsville, AL, United States

**Keywords:** *Morchella conica* SH, genomic, transcriptomic, fruiting body, endophytic bacterial communities

## Abstract

The precious rare edible fungus *Morchella conica* is popular worldwide for its rich nutrition, savory flavor, and varieties of bioactive components. Due to its high commercial, nutritional, and medicinal value, it has always been a hot spot. However, the molecular mechanism and endophytic bacterial communities in *M. conica* were poorly understood. In this study, we sequenced, assembled, and analyzed the genome of *M. conica* SH. Transcriptome analysis reveals significant differences between the mycelia and fruiting body. As shown in this study, 1,329 and 2,796 genes were specifically expressed in the mycelia and fruiting body, respectively. The Gene Ontology (GO) enrichment showed that RNA polymerase II transcription activity-related genes were enriched in the mycelium-specific gene cluster, and nucleotide binding-related genes were enriched in the fruiting body-specific gene cluster. Further analysis of differentially expressed genes in different development stages resulted in finding two groups with distinct expression patterns. Kyoto Encyclopedia of Genes and Genomes (KEGG) pathway enrichment displays that glycan degradation and ABC transporters were enriched in the group 1 with low expressed level in the mycelia, while taurine and hypotaurine metabolismand tyrosine metabolism-related genes were significantly enriched in the group 2 with high expressed level in mycelia. Moreover, a dynamic shift of bacterial communities in the developing fruiting body was detected by 16S rRNA sequencing, and co-expression analysis suggested that bacterial communities might play an important role in regulating gene expression. Taken together, our study provided a better understanding of the molecular biology of *M. conica* SH and direction for future research on artificial cultivation.

## Introduction

Medicinal mushrooms have become an attractive option for hygienic food or as a source of developing pharmaceuticals and nutraceuticals (Wasser, 2017). Medicinal properties for human health may be due to the various cellular components and secondary metabolites of the fruiting body (Martin et al., 2010; Chen et al., 2012; Lo et al., 2012). True morels (*Morchella* spp.) are ascomycetous fungi with high reputation for their edibility and appearance, which is similar to “a sponge on a stick” (Vieira et al., 2016). Taxonomically, *Morchella* is a well-defined genus in the class of Pezizomycetes, which includes species that typically produce large, fleshy, stipitate fruiting bodies. These fruiting bodies have rib-shaped, normally a honeycomb-shaped, bottle cap and is popular as an edible fungus. Morels commonly grow in a wide variety of habitats and are morphologically variable (Wipf et al., 1996; Dalgleish and Jacobson, 2005). Due to their unique flavor and nutritional value, these morels are used in soups and gravies, as a source of medicinal adaptogens, immunostimulants, and potential antitumor agents (Fu et al., 2013; Tietel and Masaphy, 2018). It is also widely used in traditional Chinese medicine to treat indigestion, excessive phlegm, and shortness of breath (Meng et al., 2010; Fu et al., 2013).

To date, differentially expressed genes (DEGs) between the mycelium and fruiting bodies were identified by high-throughput sequencing in several mushrooms, including *Agrocybe aegerita*, *Auricularia polytricha*, *Cordyceps militaris*, *Ganoderma*, and *Lentinula edodes*, and the morphological changes during the fruiting body formation were regulated by the transcription levels of development-specific genes (Song et al., 2018). Recently, the whole-genome sequence analysis of *Morchella importuna* and *Morchella sextelata* was carried out (Liu et al., 2018; Murat et al., 2018; Mei et al., 2019), but the genome and ecology of *Morchella* are not well understood. Besides, a series of studies indicate that the bacterial communities are associated with morel development. For example, *Pseudomonas* has been found to be a beneficial associate of morels previously (Pion et al., 2013); *Pedobacter*, *Pseudomonas*, *Stenotrophomonas*, and *Flavobacterium* were found to comprise the core microbiome of soil for *M. sextelata* ascocarps (Benucci et al., 2019). However, most bacterial community experiments were performed on the soil sample, not fruiting body samples. In our study, we conducted genome and transcriptome analysis of *Morchella conica* SH, as well as the endophytic bacterial communities, which will facilitate a better understanding of the molecular biology of *M. conica* SH.

## Materials and Methods

### Strains and Culture Conditions

Natural *M. conica* SH samples were collected from a local farm affiliated with Yunnan Academy of Agricultural Sciences, Yunnan Province, China. Fruiting bodies from different development stages (42–84 days) were randomly selected. After thorough washing with double distilled water, the fruiting bodies were immediately stored in sterilized valve bags and flash frozen in liquid nitrogen. Mycelia isolated from fruit bodies were cultivated and maintained on YPD agar (0.2% yeast extract, 0.2% peptone, 2% dextrose, and 2% agar) at 25°C in the dark.

### Genome Sequencing and Assembly

High-quality genomic DNA (gDNA) was extracted from 8-day mycelia of *M. conica* SH using a modification of the cetyltrimethylammonium bromide (CTAB) method established by Bao et al. (2013). Optimal quality and quantity of the extracted gDNA were verified using NanoDrop (Thermo Scientific, Wilmington, DE, United States) and Qubit 2.0 fluorometer (Invitrogen, Grand Island, United States). The gDNA library for sequencing was then prepared with TruSeq DNA sample Prep Kit (Illumina, United States) according to the manufacturer’s instructions, and the library quality was performed on Agilent 2100 Bioanalyzer (Agilent, United States) and Quantiflour-ST fluorometer (Promega, United States). Whole-genome shotgun sequencing of *M. conica* SH was performed using an Illumina MiSeq platform (Personalbio Co., Shanghai, China). Reads with low quality (> 50% bases with *Q*-value ≤ 8) and Ns > 10% in read length and adapter contaminations were removed from raw data to obtain clean reads. Clean reads were then assembled using Newbler version 2.8. Gap filling was done by GapCloser v1.12 module from SOAPdenovo2 (Luo et al., 2012). The integrity of assembly was assessed using Benchmarking Universal Single-Copy Orthologs (BUSCO; Simao et al., 2015). Mitochondrial assembly was screened out from the assembly result by comparing with mitochondrial DNA of other species in *Morchella* using BLASTX. The Whole Genome Shotgun project has been deposited at DDBJ/ENA/GenBank under the accession VOVZ00000000. The version described in this paper is VOVZ01000000.

### Gene Prediction and Functional Annotation

RepeatMasker (open) v4.06^1^ and the RepBase library^2^ (Bao et al., 2015) were used to mask genomic assembly scaffolds. The tRNAs were predicted by tRNAscan-SE (Lowe and Eddy, 1997) with eukaryote parameters. The rRNA sequences were aligned using BLASTN with *E*-value 1e^–5^ to identify the rRNA fragments. The snRNA genes were predicted by Erpin v5.4 software (Gautheret and Lambert, 2001) based on the Rfam database.

Genes were predicted using Augustus (Stanke et al., 2008) trained on core eukaryotic genes (CEGs) identified by Core Eukaryotic Genes Mapping Approach (CEGMA) (Parra et al., 2007). Gene model predictions were compiled using EVidenceModeler (EVM; Haas et al., 2008) including evidence from sequenced transcripts from *M. conica* SH assembled into contigs using PASA (Haas et al., 2011). A summary of reported *Morchella* genomes is listed in Table 1. Multiple sequence alignments between the *M. conica* SH and *M. importuna* CCBAS932 (JGI project ID: 1023999) genomes were performed using Nucmer (Kurtz et al., 2004) and visualized by Circos (Krzywinski et al., 2009).

**TABLE 1:** Statistics of the genome assembly of *Morchella*.

Assembly features	*M. conica* SH	*M. importuna* CCBAS932 (Murat et al., 2018)	*M. importuna* M04M24 (Liu et al., 2018)	*M. importuna* M04M26 (Liu et al., 2018)
Estimated genome size (Mb)	55.97	54.56	54.74	54.15
G+C content (%)	47.5	47.3	47.3	47.3
Number of contigs	2,692	1,793	763	111
Number of scaffolds	207	540	394	106
Main genome contig sequence total (Mb)	51.7	48.2	48.7	51.1
Main genome scaffold sequence total (Mb)	51.7	48.2	49	51.1
Main genome scaffold N50 (Kb)	750.2	600	653.8	978.7
**Gene models**				
Number of predicted genes	9,676	11,600	11,519	11,770
Average exons length (bp)	556	413	165	169
Average exon frequency (exons/gene)	4	3.46	3.96	3.99
Average intron length (bp)	103	100	191	191
Coverage of genome-coding region (complete/partial)^a^	95.1%	98%	91.1%	93%

*a Genome-coding region coverage was estimated by Benchmarking Universal Single-Copy Orthologs (BUSCO; Simao et al., 2015).*

Annotation of predicted gene models was performed by similarity searches using BLASTp (*E*-value: 1e^–5^) based on Swiss-Prot, GenBank’s database of nonredundant proteins, and TrEMBL database (Bairoch and Apweiler, 2000). SignalP 3.0^3^ was used to predict putative secreted proteins. KEGG Automatic Annotation Server (KAAS) analysis was used for KEGG ortholog mapping (Moriya et al., 2007), and InterProScan (Goujon et al., 2010) was used for domain annotation. Proteins were annotated using BLAST2go (Conesa et al., 2005) on the NCBI nonredundant protein database. Predicted proteases were obtained by BLASTp based on MEROPS database (Rawlings et al., 2010) and Pfam databases, respectively. To identify secondary metabolite gene clusters, the genome sequence was analyzed by online program natural product search engine (Li et al., 2009) and antiSMASH (Blin et al., 2013).

### Orthology and Phylogenetic Analysis

*M. conica* SH and the other 15 fungi, *Magnaporthe oryzae* (Choi et al., 2015), *Neurospora crassa* FGSC 73 (Baker et al., 2015), *Nectria haematococca* (Coleman et al., 2009), *Sclerotinia sclerotiorum* (Amselem et al., 2011), *Botrytis cinereal* BcDW1 (Blanco-Ulate et al., 2013), *Stagonospora nudorum* (Hane et al., 2007), *Aspergillus niger* ATCC 1015 (Andersen et al., 2011), *Arthrobotrys oligospora* (Yang et al., 2011), *Pyronema confluens* (Traeger et al., 2013), *Tuber melanosporum* Vittad (Martin et al., 2010), *Ganoderma lucidum* (Chen et al., 2012), *Saccharomyces cerevisiae* YJM789 (Wei et al., 2007), *Saccharomyces pombe* (Rhind et al., 2011), *M. sextelata* (Mei et al., 2019), and *M. importuna* (Liu et al., 2018), were chosen for orthology analysis. All protein-coding genes were analyzed by OrthoMCL (Li et al., 2003) using an all-versus-all BLASTp. Genes share more than 50% sequence identity, and 50% covery was assigned into clusters. For putative orthologs or paralogs, the *p*-value cutoff was 1e^–5^. The Markov cluster (MCL) algorithm was used to assign proteins into families (Enright et al., 2002). Then, single-copy orthologs were selected to align using MUSCLE (Edgar, 2004), trimmed with Gblocks (Castresana, 2000). The maximum likelihood analysis was built by RAxML (Stamatakis, 2006) under JTT model and 100 bootstrap replicates (Burleigh et al., 2006). The divergence time between species was estimated by the Langley–Fitch method with r8s (Sanderson, 2002) calibrated based on the origin of Ascomycota at 500–600 million years ago (MYA; Taylor and Berbee, 2006).

### Collinearity Analysis

All-versus-all BLASTp was performed to identify paralogous or orthologous gene pairs, and the blast result associated with *M. conica* SH annotation file was processed with MCScanX to identify collinear blocks (Wang et al., 2012). The results were visualized using Circos plot (Krzywinski et al., 2009).

### Other Protein Families

Putative enzymes involved in carbohydrate utilization were predicted by a combination of BLASTp and HMMER (Mistry et al., 2013) searches based on carbohydrate-active enzyme database (Cantarel et al., 2009). Lipases were predicted by using BLASTp based on the Lipase Engineering Database (Fischer and Pleiss, 2003). Additionally, transporters, protein kinases, transcription factors (TFs), and lignocellulose-active proteins were classified by BLASTp (*E*-value cutoff 1E^–10^) based on Transporters Classification Database^4^ (Saier et al., 2006), KinBase^5^, Fungal Transcription Factor Database (FTFD^6^; Park et al., 2008), and Characterized Lignocellulose-Active Proteins of Fungal Origin Database (mycoCLAP^7^; Strasser et al., 2015), respectively.

### Transcriptome Analysis

Gene expression profiles were evaluated in different development stages of *M. conica* SH. X0 is the free-living mycelium (FLM) stage, and X1, X2, X3, and X4 samples represent 1, 2, 4, and 6 weeks of fruiting body, respectively. Three biological replicates for each sample were analyzed.

Total RNA was extracted from the whole fruiting body using RNeasy Mini Kit (Qiagen, United States), according to the manufacturer’s protocols. Then, ND-2000 spectrophotometer (NanoDrop Technologies) and Bioanalyzer 2100 (Agilent Technology, United States) were used to evaluate the quantity and quality of RNA. RNA libraries were constructed and size-selected using VAHTS mRNA-seq v2 Library Prep Kit for Illumina (Vazyme, Nanjing, China) and AMPure XP Beads (Beckman, Germany), respectively. The libraries were amplified by PCR (15 cycles) and sequenced on HiSeq X platform at Shanghai Sangon Biotechnology Corporation.

Raw sequencing reads of Fastq files were filtered using seqtk^8^, which masked bases with *Q*-scores < 20. The clean data were obtained by removing reads containing adapters, ribosome RNA reads, and low-quality reads from raw data and then mapped to reference genome using Hisat2 version 2.0.4 (Kim et al., 2015). Those uniquely mapped reads were retained for read counting against the annotated genes using StringTie version 1.3.0 (Pertea et al., 2015). The expression level of the unique gene was calculated as fragments per kilobase of exon model per million fragments mapped (FPKM; Mortazavi et al., 2008) by RSEM tool (Li and Dewey, 2011). The edgeR was then used to identify DEGs (Robinson et al., 2010).

Co-expression networks were constructed using the pairwise Pearson correlations between DEGs. Louvain method for community detection was employed to part the network into modules of genes displaying similar expression profiles. To test associations between microbial taxa and modules, we used the MaAsLin2 (Morgan et al., 2012) to assess the relationship between the top 10 most abundant *Morchella-*associated microbes and co-expression network modules. Co-expression networks were visualized in Cytoscape (Shannon et al., 2003).

### Amplicon Sample Preparation for 16S rRNA

Microbial community gDNA was extracted from different development stages of *M. conica* SH fruiting body using the FastDNA^®^ Spin Kit for Soil (MP Biomedicals, GA, United States) according to manufacturer’s instructions. The DNA extract was checked on 1% agarose gel, and DNA concentration and purity were determined with NanoDrop 2000 UV-vis spectrophotometer (Thermo Scientific, Wilmington, DE, United States). The hypervariable region V3–V4 of the bacterial 16S rRNA gene was amplified with primer pairs 338F (5′-ACTCCTACGGGAGGCAGCAG-3′) and 806R (5′-GGACTACHVGGGTWTCTAAT-3′) by an ABI GeneAmp^®^ 9700 PCR thermocycler (ABI, CA, United States). The PCR amplification of 16S rRNA gene was performed as follows: initial denaturation at 95°C for 3 min, followed by 27 cycles of denaturing at 95°C for 30 s, annealing at 55°C for 30 s and extension at 72°C for 45 s, and single extension at 72°C for 10 min, and end at 4°C. The PCR mixtures contain 5 × *TransStart* FastPfu buffer 4 μl, 2.5 mM dNTPs 2 μl, forward primer (5 μM) 0.8 μl, reverse primer (5 μM) 0.8 μl, *TransStart* FastPfu DNA Polymerase 0.4 μl, template DNA 10 ng, and finally ddH_2_O up to 20 μl. PCR reactions were performed in triplicate. The PCR product was extracted from 2% agarose gel and purified using the AxyPrep DNA Gel Extraction Kit (Axygen Biosciences, Union City, CA, United States) according to the manufacturer’s instructions and quantified using Quantus^TM^ Fluorometer (Promega, United States).

### 16S rRNA Gene Sequencing and Bioinformatic Analysis

Purified amplicons were pooled in equimolar and paired-end sequenced on an Illumina MiSeq PE300 platform (Illumina, San Diego, CA, United States) according to the standard protocols by Majorbio Bio-Pharm Technology Co. Ltd. (Shanghai, China). The raw 16S rRNA gene sequencing reads were demultiplexed, quality-filtered by fastp version 0.20.0, and merged by FLASH version 1.2.7 with the following criteria: (i) the 300-bp reads were truncated at any site receiving an average quality score of < 20 over a 50-bp sliding window, truncated reads shorter than 50 bp were discarded, and reads containing ambiguous characters were also discarded; (ii) only overlapping sequences longer than 10 bp were assembled according to their overlapped sequence. The maximum mismatch ratio of overlap region is 0.2. Reads that could not be assembled were discarded; (iii) Samples were distinguished according to the barcode and primers, and the sequence direction was adjusted according to exact barcode matching and two nucleotides mismatch in primer matching.

Operational taxonomic units (OTUs) with 97% similarity cutoff were clustered using UPARSE version 7.1, and chimeric sequences were identified and removed. The taxonomy of each OTU representative sequence was analyzed by RDP Classifier version 2.2 based on the 16S rRNA database using a confidence threshold of 0.7.

### Quantitative Real-Time PCR Analysis

Quantitative real-time PCR (qRT-PCR) was used to validate the assembled transcriptome from the RNA sequencing (RNA-seq) experiment. Total RNA was extracted from the mycelium (X0) and the whole fruiting body (X1, X2, X3, X4). RNA was isolated by using an RNA Purification Kit (TIANGen Biotech, Beijing, China). Then, total RNA (2 μg) was reverse-transcribed to cDNA by reverse transcription mix (Promega). qRT-PCR reactions were performed on an ABI 7500 system (ABI, CA, United States). The primers used in qRT-PCR are summarized in Supplementary Table 24. All qRT-PCR experiments were performed in triplicate using independent samples (Meng et al., 2019). The expression levels were determined by the 2^–ΔΔCt^ method using *CYC3* gene as internal control genes for normalization (Zhang et al., 2018).

### Availability of Data

Raw transcriptomic data have been deposited in the NCBI with the SRA project ID PRJNA733830.

## Results

### Genome Sequencing, Assembly, and Genomic Features

The genome sequence of *M. conica* SH was carried out by the whole-genome shotgun sequencing strategy. A total of 7.98 Gb raw data were generated for paired-end reads. After quality filtering, 6.38-Gb clean data with 113.97 × coverage obtained (Supplementary Table 1), and 51.7-Mb genome assembly with 1.49% repeat content was ultimately used for further analysis (Supplementary Table 2). Based on K-mer statistics, the *M. conica* SH genome was estimated to be 55.97 Mb (Supplementary Table 3), indicating that 92.4% of the genome was captured in scaffolds. The assembly comprises 2,692 contigs (N50 = 41.7 kb) and 207 scaffolds (N50 = 0.75 Mb). According to BUSCO (Simao et al., 2015), 98.75% of the fungal single-copy orthologs were found in gene repertoires, suggesting that the assembly of *M. conica* SH is highly accurate. In total, 9,676 protein-coding genes were predicted, with an average transcript length of 2,336 bp and an average exon number of 4 (Table 1). The functions of these genes were predicted by different databases. Compared with other databases, large proportion genes (79.7%) were enriched in NR (Supplementary Table 4). We also found that our data are comparable to the assembly statistics in the *M. importuna* strains (Table 1). Furthermore, we assembled the mitochondrial genome into one scaffold with a length of 262,110 bp (Supplementary Result 1). The majority (72%) of these genes are hypothetical proteins with unknown functions. However, 590 secreted proteins (6%) were found in *M. conica* SH; the rest are glycoside hydrolases and proteins involved in growth and utilization of carbohydrates.

### Phylogenetic Analysis of Evolutionary Relationship Between *M. conica* SH and Other Related Fungal Species

**FIGURE 1:**
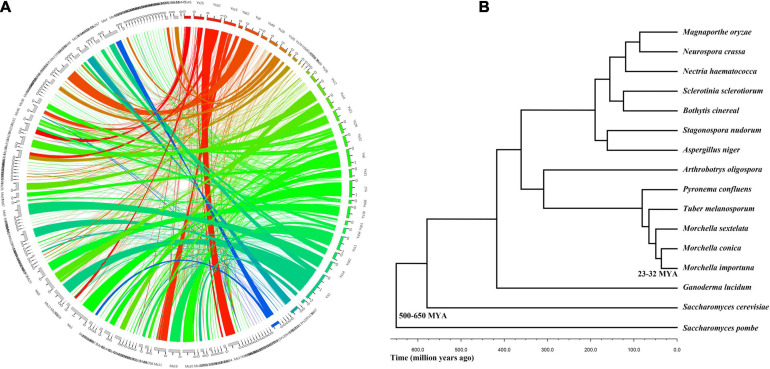
Evolutionary relationship between *Morchella conica* SH and other related fungal species. **(A)** Genome synteny in *Morchella*. Circos plot showing syntenic regions in two *Morchella* species (*M. conica* SH in colored blocks, *Morchella importuna* in gray blocks). One syntenic block is considered only if at least two orthologous genes are present in the same order. The yellow, green, and blue links correspond to syntenic blocks composed of > 10, 5–10, and < 5 genes, respectively. **(B)** The phylogenetic tree of *Morchella* and 13 other species in the context of fungi.

In order to identify the relationship between *M. conica* SH and other related fungal species, we compared the genome structures of the 207 scaffolds in *M. conica* SH assembly with that of the 504 scaffolds in the *M. importuna* CCBAS932 assembly (Murat et al., 2018; Figure 1A). A total of 118 matching scaffold block pairs ranging from 102 Kb to 1.4 Mb were identified between two genomes. Among those blocks, 31 contained inversions and translocations, 23 contained only inversions, 10 were completely synthetic with no inversion or translocation, and 38 contained only translocations. When comparing the predicted proteins, 7,713 of *M. conica* SH showed significant similarities (> 79%) to known proteins from *M. snyderi* DOB2414, *M. importuna* SCYDJ1-A1, and *M. importuna* CCBAS932 (Liu et al., 2018; Murat et al., 2018). Additionally, we found that most putative homologs were identified in the Pezizomycotina, and there is high sequence similarity between 4,630 predicted proteins and that from *T. melanosporum* (Martin et al., 2010).

To investigate the evolutionary status of *M. conica* SH, multigene families were predicted from proteins in *M. conica* SH and 14 other related representative species using the MCL algorithm (Enright et al., 2002). Finally, 672 single-copy orthologous genes were obtained and used to construct a phylogenetic tree (Supplementary Table 5). The phylogeny was inferred using RAxML version 7.1.0 with the PROTGAMMAJTT model and 100 bootstrap replicates. The divergence time of the *Morchella* was estimated at 25–32 MYA using r8s (Sanderson, 2002) calibrated with divergence times of 500–600 MYA for Ascomycota. *M. conica and M. importuna* were gathered into one cluster and separated from *M. sextelata.* As the sister taxon of *T. melanosporum* (diverged about 25–32 MYA), *M. conica* was significantly diverged from *P. confluens* (57–65 MYA), which belonged to Pezizomycetes (Figure 1B).

### Functional Characterization of *M. conica* SH

In order to characterize the function of predicted genes, we compared the genes involved in specific pathways among *M. conica* SH and nine other Pezizomycetes, including five saprotrophs (*M. importuna*, *Ascobolus immersus*, *Asscodesmis nigricans*, *Plectania confluens*, and *Sarcoscypha coccinea*) and four ectomycorrhizal (ECM) fungi (*Tuber borchii*, *Tuber melanosporum*, *Terfezia boudieri*, and *Choiromyces venosus*), as well as 12 additional taxonomically and ecologically distinct fungi, including other ECM, saprotrophs, and pathogens (Supplementary Table 6). Genes belonging to four functional clusters were annotated.

#### Repertoire of Degrading Enzymes

**FIGURE 2:**
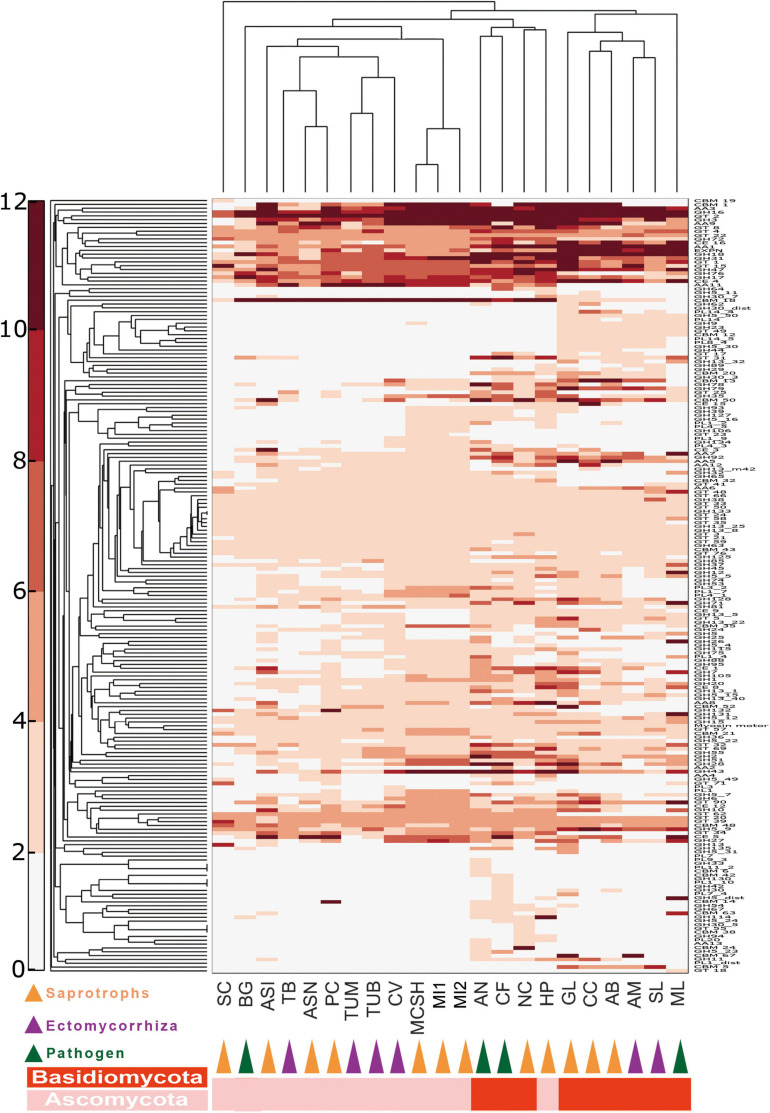
Double hierarchical clustering of the carbohydrate-active enzyme (CAZyme)-coding gene numbers in representative fungal genomes. A double hierarchical clustering of the number of CAZyme-coding genes for each of the fungal species was performed using the MatLab software. The Euclidian distance between gene counts was used as distance metric, and a complete linkage clustering was performed. The relative abundance of genes is represented by a color scale (on the left) from the minimum (white) to the maximum (red) number of copies per species. See Supplementary Table 6 for full names of species and lifestyles.

Lignin is an aromatic macromolecule that protects the plant cellulose and hemicellulose against biodegradation. To reveal the carbohydrate-digesting capabilities of *M. conica* SH, we analyzed genes involved in the degradation of polysaccharides, proteins, and lipids (Figure 2 and Supplementary Tables 7–13). Overall, 232 putative proteases were identified in *M. conica* SH genome, the majority (171) were also found in *M. importuna* CCBAS932 according to the MEROPS database^9^ (Supplementary Table 7). Among them, 18% of proteases identified in *M. conica* SH contained signal peptides, the same as the pathogenic fungi (Supplementary Table 8). Additionally, *P. melastoma* had more proteases and secreted proteases than other species (Supplementary Tables 7, 8), while *A. bisporus* had the least. Compared with other members in Pezizomycetes, aspartic peptidases and threonine peptidases were significantly enriched (*t*-test, *p* < 0.005) in *Morchella* genomes. Notably, Pezizomycetes genomes include nearly twice the median number of lipase (Supplementary Table 9). In the *M. conica* SH genome, a total of 90 lipases from microsomal hydrolases family (GX-abH09) were annotated, which is significantly more (*t*-test, *p* < 0.005) than the other species. However, no difference was observed in the profile of lipases and secreted lipases among Pezizomycetes (Supplementary Tables 9, 10).

Ectendomycorrhiza and Ectomycorrhiza (ECM) fungal genomes contained the lowest number of carbohydrate-active enzyme (CAZyme) genes, while saprotrophic fungi contain more CAZyme genes (Supplementary Tables 11, 12). Specifically, Basidiomycota harbors more redox enzymes acting in conjunction with CAZymes (AAs) and carbohydrate esterases (CEs), while *Morchella* has more polysaccharide lyases (PLs) than any other fungi. In addition, saprotrophic species have nearly 50% more CAZyme genes compared with other Pezizomycetes, which possess a different lifestyle (Supplementary Figure 1A). The profiles of CAZymes among *Morchella* species are almost identical. CAZyme profiles revealed that *M. conica* SH had 177 glycoside hydrolases (GHs), 21 PLs, and 26 CEs (Supplementary Table 1A). Additionally, one cuprum peroxidase was found in the genome.

CAZyme families involved in lignocellulose degradation were analyzed in our study. Compared with ECM species, saprotroph genomes encoded a greater set of genes coding for lignocellulose oxidoreductases (Supplementary Figure 1B and Supplementary Table 13), such as cellobiose dehydrogenases (AA3), iron reductases (AA8), and lytic polysaccharide monooxygenases involving cleavage of cellulose (AA9), degradation of cellulose (GH 5_7, GH5_16, GH 6 and GH 7, GH 45 and GH 115), hemicellulose and/or pectin (GH 27, GH 28, GH 39, GH 43, GH 51, GH 53, GH 88, GH 93, PL 1, and PL 3), also glucans (GH 15 and GH 71). Among Pezizomycetes, according to BLAST analysis in lignocellulose-active protein database (see text footnote 7), 18 CAZyme families involving lignocellulose degradation were significantly enriched in *Morchella* (Supplementary Table 13). They are involved in the degradation of cellulose (GH 115), pectin or/and hemicellulose (GH 6, GH 26, GH 27, GH 30, GH 43, GH 79, and PL 1), and glycan (GH 12, GH 13, and GH 43).

#### Genes Associated With Secondary Metabolism

Plant-associated fungi produce secondary metabolites that are ecologically and nutritionally benefit to plants (Spatafora and Bushley, 2015). The core genes associated with putative secondary metabolite biosynthesis are often in gene clusters, including polyketide synthases (PKSs), non-ribosomal peptide synthetases (NRPSs), members of the 4-dimethylallyltryptophan synthetase (DMATS), and terpene synthases (TSs). Most Pezizomycetes do not contain DMATS genes compared with other types of fungi. However, *P. confluens* contains a large amount of secondary metabolism genes (Supplementary Table 14).

There are seven NRPS gene clusters, two PKS-like gene clusters, and one TS gene cluster in the *M. conica* SH genome. One of the NRPS proteins, Scaffold26.t84, has a typical extracellular siderophore domain (Supplementary Figure 2A), while another NRPS protein (Scaffold11.t41) harbors an adenylation domain (A_NRPS; cd05930), and four NRPS proteins (Scaffold7.85, Scaffold16.t118, Scaffold10.t106, and Scaffold24.t128) contain Acyl-CoA synthetase domain (CaiC; COG0318; Supplementary Figure 2B). The type I PKS gene (*scaffold7.86*) was adjacent to another putative NRPS gene (*scaffold7.85*), and the Acyl transferase domain (PksD; COG3321) was found in its product (Supplementary Figure 2C). The remaining NRPS genes have no homology to NRPS with known functions.

#### Transporters

In our study, a total of 269 transporter families detected in all the fungi were analyzed. As observed, 162 families were commonly presented in all species (Supplementary Table 15). Bioinformatic prediction was performed by TransportDB 2.0 (Elbourne et al., 2017), and transporter genes were clustered according to their types or functions (Supplementary Table 15). As expected, the most commonly enriched family was the major facilitator superfamily (MFS), followed by the ATP-binding cassette (ABC) and mitochondrial carrier (MC). On the other hand, BLAST analysis for the transporter classification database (TCDB; Saier et al., 2006) showed that 1,213 gene products in *M. conica* SH genome have homologs with > 30% identity in TCDB (Supplementary Table 16), and the most abundant is the MFS Superfamily (2.A.1). Compared with other Pezizomycetes, the membrane attack complex/perforin family (MACPF, 1.C.39) and the ion-translocating microbial rhodopsin family (MR, 3.E.1) are missing in *Morchella*. We also found that some transporter families are strain-specific, for example, the profiles of SH strain and CCBAS932 strain are obviously different (Supplementary Table 16). Interestingly, two transporter families, the sorting nexin27 (SNX27)-retromer assembly apparatus for recycling integral membrane proteins (SNX27-RetromerAA, 9.A.3) and the ankyrin family (8.A.28), were expanded, and the expansion is also found in *C. venosus* genome. However, little is known about its function in fungi. The putative ductin channel family transporter (9.A.16) was only encoded by *Morchella* genomes, and an adiponectin family transporter (8.A.94) was unique in *M. conica* SH genome. In addition, BLAST result showed that the collagen-like protein family (PFAM01391, identity > 50%) has homology in *M. conica* SH genome, which is related to cell adhesion, invasion, and intracellular signaling in pathogens (Humtsoe et al., 2005).

#### Signal Transduction

Overall, CMGC (cyclin-dependent kinase, mitogen-activated protein kinase, glycogen synthase kinase, CDC-like kinase), calcium/calmodulin-dependent kinase (CAMK), and STE (homologs of the yeast *STE7, STE11, and STE20* genes) and AGC (protein kinase A, G, and C) seem to be the most common clades. Histidine kinase family was expanded in most Pezizomycetes, followed by CAMK family RAD53, which has a similar pattern in other fungi (Supplementary Table 17). Interestingly, kinase family CHK1 was the only family commonly seen in saprotrophic fungi rather than ECM fungi (*t*-test, *p* < 0.05). Among Pezizomycetes, ECM fungus *T. melanosporum* had the largest number of kinases (193 kinases). Several kinase families were abundant in *Morchella* species, such as AGC, CAMK, and STE families (*t*-test, *p* < 0.05). There are 137 protein kinases and 27 other atypical kinase genes in *M. conica* SH, while 153 and 25 were detected in *M. importuna* CCBAS932 genome, respectively. *M. conica* SH has 12 histidine kinases compared with 5–14 in other fungi (Supplementary Table 17).

Gene expression is controlled by the activation of different TFs, which are essential regulatory proteins involved in a variety of cell functions. Putative TFs were identified by BLASTP analysis based on the FTFD (see text footnote 6). The candidates were classified according to Park et al. (2008) (Supplementary Table 18). In general, Pezizomycetes have more TFs than other species, especially in zinc finger classes. However, there is no significant difference in the distribution of different classes within Pezizomycetes (Supplementary Figure 3). In *M. conica* SH, 836 TFs were predicted, including all major classes of fungal TFs, Zn2Cys6, Helix-turn-helix, AraC type, Winged helix repressor DNA-binding, and C2H2 zinc finger. Eighty-nine predicted *M. conica* SH proteins were similar to the characteristic TFs of *T. melanosporum* (BLASTp, *E*-value < 1e^–50^). Moreover, according to different cell functions, putative *Morchella* TFs were divided into five groups (Supplementary Table 19).

Additionally, the single mating-type locus in unifactorial *Agaricomycetes* was also found in the *M. conica* SH genome (*scaffold86.t1*). Individuals of heterologous fungi usually contain a *MAT1* idiomorph (Turgeon and Yoder, 2000). In this study, the *MAT1* gene in *M. conica* SH genome was homologous with *MAT1-1*(EAA35088) gene of *N. crassa*.

### Comparative Transcriptome Analysis

**FIGURE 3:**
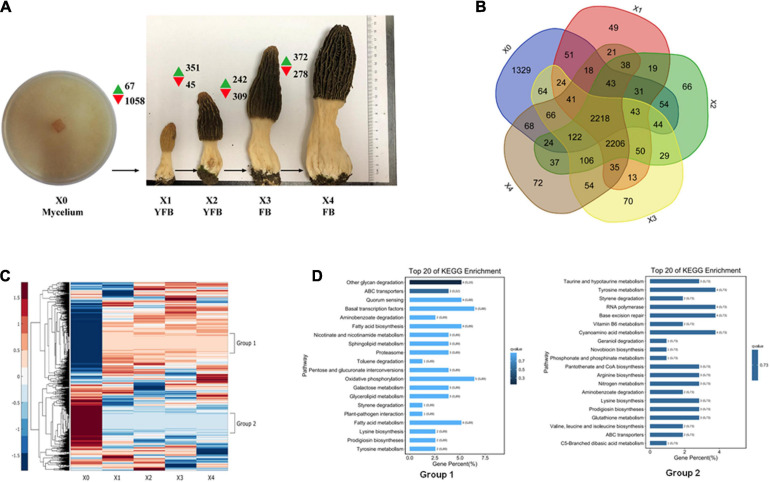
Expression analysis of developmental stages of *Morchella conica* SH. **(A)** Gene expression map of five developmental stages. Green and red arrows denote the numbers of significantly up- and downregulated genes [*p* < 0.05, fold change (FC) > 4], respectively. The development of X0 (mycelium), X1–X2 young fruiting body (YFB), and X3–X4 fruiting body (FB) is shown. Each sample was sequenced in three biological replicates. **(B)** Venn plot shows the number of detected genes overlapping in the compared developmental stages. **(C)** Heatmap shows the differentially expressed genes (DEGs) during the five development stages of *M. conica* SH; group 1 and group 2 represent distinct expression patterns. **(D)**. Kyoto Encyclopedia of Genes and Genomes (KEGG) enrichment of genes in group 1 and group 2.

The *M. conica* SH strain of the free-living mycelium (stage X0) and four different development stages of the fruiting body (X1–X4) were used for transcriptome analysis (Figure 3A and Supplementary Table 20). In total, 7,555 of 9,203 predicted genes were expressed in *M. conica* SH. As shown, 2,218 genes were stably expressed at each stage, 1,329 genes were specifically expressed at X0 stage, and 2,206 genes were commonly expressed in the fruiting body (Figure 3B). We then compared the genes expressed in the mycelium and fruiting body and found that 2,233 genes were expressed in both stages, while 1,329 and 2,796 genes were specifically expressed in the mycelium and fruiting body, respectively. The Gene Ontology (GO) enrichment showed that RNA polymerase II transcription activity-related genes were enriched in the mycelium-specific gene cluster, and nucleotide binding-related genes were enriched in the fruiting body-specific gene cluster (Supplementary Figure 4).

**FIGURE 4:**
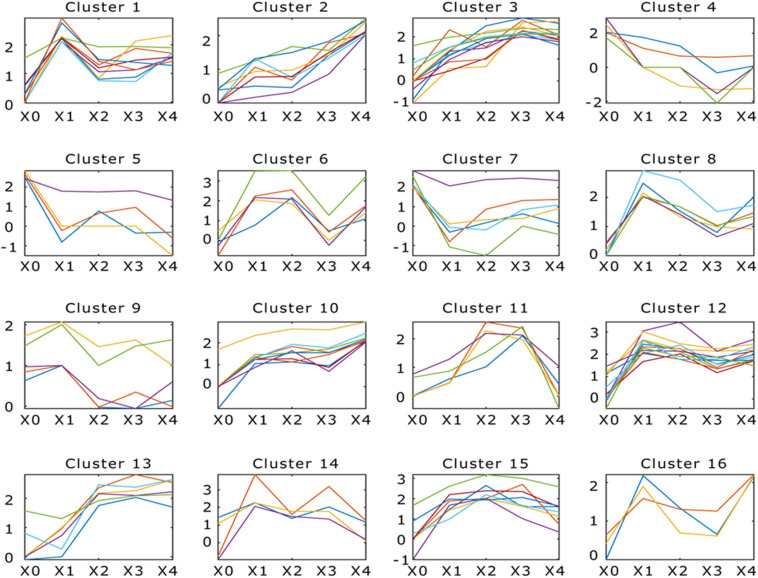
Dynamic progression of *Morchella conica* SH transcriptome in different development stages. K-means clustering showing the expression profile of the *M. conica* SH transcriptome. Sixteen clusters were identified and presented along the five stages (X0, X1, X2, X3, and X4) from 9,203 differentially expressed genes; genes of each cluster were listed in Supplementary Table 21.

The DEGs (adjusted *p* < 0.05, fold change ≥ 2) between different development stages were identified. Comparing between X0–X1, X1–X2, X2–X3, and X3–X4 growth stages, 1,125, 396, 551, and 650 protein-coding genes were significantly up- or down-regulated (*p* < 0.05, fold change ≥ 2), respectively (Figure 3 and Supplementary Table 20). To study the function of the DEGs in the five development stages, GO terms and KEGG Orthology (KO) terms were used to classify genes to functional categories and pathways. DEGs were grouped into 16 clusters by parasitic dynamics using the K-Means clustering algorithm (Figure 4). Genes with a high expression level in the mycelium were enriched in clusters 4, 5, and 7, and GO analysis displays that they were involved in cellular component and transport, while genes with high expression levels in the fruiting body were enriched in clusters 2, 3, 10, and 13 and related to phospholipid metabolic process and kinase activity (Supplementary Table 21). The expression pattern was illustrated by Heatmap. Two groups of genes showed distinct expression patterns (Figure 3D). Genes belonging to group 1 were significantly upregulated in the fruiting body (X1–X4), and KEGG analysis suggested that they were mainly involved in other glycan degradation and ABC transporters, while genes belonging to group 2 were dramatically downregulated in the fruiting body and were mainly enriched in taurine and hypotaurine metabolism, tyrosine metabolism, and styrene degradation.

In order to identify core genes involved in biological processes during the development of the fruiting body, we compared transcriptome profiles of undifferentiated mycelium grown on agar medium with the fruiting body at different stages. Of the 7,555 transcripts detected in the fruiting body, 226 (3%) were significantly upregulated (*p* < 0.05) in all the stages of the fruit body. Among the 50 most highly induced genes in the fruiting body, transporters, fungal transcriptional factors, and kinases were among the most highly induced genes (Supplementary Table 22). Several transcripts coding for proteases (M24A, T01A, I87, M18, and T01X families) were upregulated transcripts during X0–X1, and the most upregulated protease was one of T01A family (Protein ID Scaffold23.t27; Supplementary Table 23), showing a 300-fold induction in the fruiting body. Ten proteases (C26, S08A, M24X, T03, A01A, C12, M28X, and M41 families) were downregulated in the fruiting body, including the gene encoding deubiquitinating enzyme Uch2 (*scaffold2.t334*) from C12 family. Among the lipases, an Alpha esterase gene (*scaffold15.t73*) from abH01 family was significantly upregulated in the fruiting body, while *scaffold47.t53* and *scaffold39.t45* showed a 9,000-fold and 100-fold induction in the mycelium.

### Validation of Gene Expression by qRT-PCR

To validate the RNA-Seq results, six genes were randomly selected from the significant gene expression patterns of group 1 and group 2 and analyzed by qRT-PCR. The CYC3 gene was used as an internal reference gene for qRT-PCR analyses, which has been evaluated as the most stable gene in different development stages (Zhang et al., 2018). For each gene, the expression count values of transcriptome data exhibited a similar expression profile compared with the qPCR results, and the RNA-seq and qRT-PCR results revealed a strong correlation with each other (Pearson correlation, *r* = 0.8715, *p* = 3.5421 E- 15), suggesting reliable expression results generated *via* RNA-seq (Supplementary Figure 6).

### Dynamic Shift of Microbial Communities in the Development of the Fruiting Body

**FIGURE 5:**
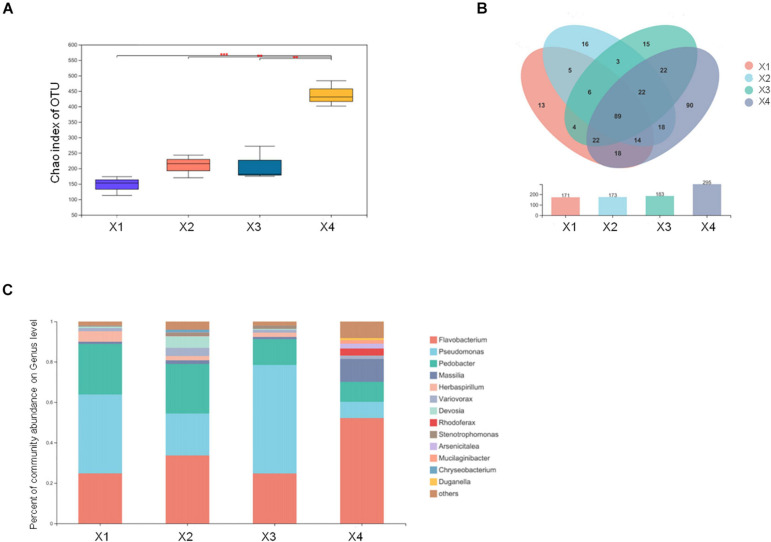
Different microbial communities in the fruiting body development stages. **(A)** The Chao index of operational taxonomic unit (OTU) levels. **(B)** Venn plot shows the number of detected OTUs of the fruiting body. **(C)** The endophytic microbial community analysis with development stages of the fruiting body.

Microbial communities can trigger the formation of primordia and the development of the fruiting body. To understand the microbial communities in *M. conica* SH, we examined the microbial communities in four development stages of the fruiting body. By detecting bacterial 16S rRNA, 515 OTUs were identified in at least one stage (Supplementary Table 25). When comparing different stages, we found that no distinct changes on the Chao index of OTU levels during X1–X3, but it was significantly increased at X4 (Figure 5A). Venn plot showed that a large number of bacterial communities (106) can be detected in the four stages, which might be the essential bacteria for the development of the fruiting body. And 18, 26, 18, and 152 specific communities were detected at the four stages, suggesting more communities might be involved in the degradation of useless substrate at the mature stage (Figure 5B). To further identify the dominant bacterial communities at each stage, we calculated the percentage of community abundance on the genus level. As observed, high abundances of *Flavobacterium*, *Pseudomonas*, and *Pedobacter* were found in the four stages, while *Massilia* and *Herbaspirillum* were highly abundant at the first three stages or mature stages, respectively (Figure 5C). As previously reported, *Flavobacterium* associated with promoting the development of its host (Chaudhary et al., 2019; Kim and Yu, 2020). *Massilia* was involved in the synthesis of multiple secondary metabolites and enzymes, phosphorus solubilization, degradation of phenanthrene, and resistance to heavy metals (Feng et al., 2016; Zheng et al., 2017). *Herbaspirillum* could fix nitrogen in rice and sugarcane (Hoseinzade et al., 2016). Our results suggested that these bacterial communities might play an important role in the development and maturity of *M. conica* SH.

**FIGURE 6:**
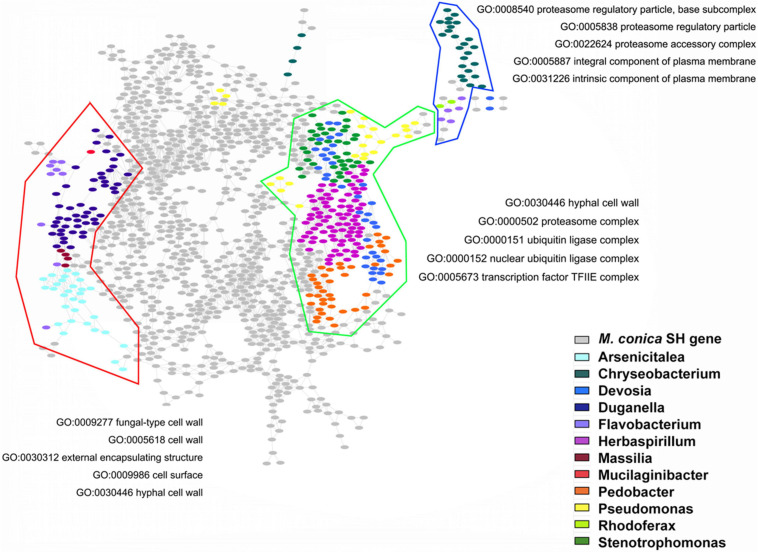
Gene Ontology (GO) functions of bacteria-regulated differentially expressed genes (DEGs). Using fruiting body-associated DEGs (gray dots), we created a co-expression network, which was partitioned into modules by network community detection (colored lines enclosed). Top five enriched GO terms were indicated for each network module. Hypergeometric tests revealed the bacteria-regulated genes (colored dots) that mapped the network.

In order to check the possible regulatory roles of bacterial communities in gene expression, we investigated the GO functions of bacteria-regulated DEGs. Three main gene clusters highly correlated with the abundance of bacterial communities were found in our analysis. GO enrichment displayed genes associated with *Arsenicitalea* and *Duganella* involved in fungal-type cell wall, cell wall, external encapsulating structure, cell surface, and hyphal cell wall; genes associated with *Herbaspirillum*, *Pedobacter*, *Devosia*, and *Stenotrophomonas* involved in hyphal cell wall, proteasome complex, ubiquitin ligase complex, nuclear ubiquitin ligase complex, and TF TFIIE complex; and genes associated with Chryseobacterium involved in proteasome regulatory particle, proteasome accessory complex, and integral and intrinsic component of plasma membrane (Figure 6). Taken together, our results suggested that endophytic bacterial communities might promote the development of the fruiting body through regulating specific gene expression in *M. conica* SH.

## Discussion

*M. conica* was not only consumed as tasteful food, health nutritional supplement for its high gastronomic quality, fatigue resistance, and gastroprotective effects but also used in traditional Chinese medicine to potentially treat indigestion, excessive phlegm, shortness of breath, and antitumor and immunomodulatory activities (Tietel and Masaphy, 2018). However, the understanding of its basic biology and genome information is very limited. In this study, we reported the complete genome blueprint of *M. conica* SH and constructed a transcriptome and endophytic bacterial community analysis of *M. conica* SH.

This study is the first genome sequencing analysis of *M. conica* SH, whose general assembly features are similar to those of other reported *Morchella* species (Table 1). Comparison between *M. conica* SH and *M. importuna* CCBAS932 in genome structures showed inversions and translocations occurring in several homologous scaffolds, suggesting that evolutionary genome rearrangement accrued between two species. The ecology of *Morchella* spp. is not well understood; some species showed a symbiotic relationship with plant root, while some were saprotrophs (Murat et al., 2018). We compared the genome content of *M. conica* SH with those of *M. importuna* CCBAS932 and 15 other Pezizomycetes. Our research revealed a similar distribution of protein families between *M. conica* SH and other saprotrophic fungi, suggesting that *M. conica* has a saprotrophic lifestyle. Many saprotrophic fungi resemble pathogens in their repertoire of degrading enzymes; the secreted proteases in pathogenic fungi are mainly involved in the infection process (Lucking et al., 2009). However, in *M. conica* SH and other saprotrophs, they are more likely to facilitate adaption on complex cultivated environments (Heneghan et al., 2009; Erjavec et al., 2012). The most abundant protease family in *M. conica* SH is serine protease, which belongs to fibrinolytic enzymes in other edible fungi. They have great potential to be developed as functional food additives for the prevention and treatment of thrombotic diseases (Liu et al., 2017). Thirteen GH families were found in *M. conica* SH, including the GH61 family. It was reported that its cellulolytic activity was weak, and it was also detected in *T. melanosporum* Vittad (Karkehabadi et al., 2008; Martin et al., 2010). Ligninolytic fungi degrade lignin using combinations of multiple isoenzymes of three heme peroxidases: lignin peroxidases, manganese peroxidases, and hybrid enzymes (Zhao et al., 2013). The genome of *M. conica* SH contains a putative manganese peroxidase and lignin peroxidase but lacks genes encoding hybrid enzymes. Many morels (*Morchella*) were strongly biased toward burned tree trunks after wildfire (Greene et al., 2010); damaged trees may require less enzymes to degrade *M. conica* SH.

The transporter system plays a critical role in fundamental cellular processes, which are distributed through membranes to deliver essential nutrients, excrete waste products, and help cell signal transduction under environmental changes. MFS and ABC are the most common types of transporters in all sequenced fungal genomes and are usually involved in the transport of different substrates (Morschhauser, 2010). ABCs are important in defending pathogens from toxic compounds produced by the host. The profile of secondary transporter genes can reflect the fungal lifestyle. For example, *Pezizomycete* genomes lack several amino acid and ion transporter genes, but they encode other families to compensate (Supplementary Table 16). Potassium ions can be transported *via* KUP (K+ uptake) and Trk (K+ transporter) families (Schlosser et al., 1995). In ECMs, many species lack KUP family proteins, but they could use Trk family K+:H+ symporter. In contrast, saprotrophic fungi are enriched in genes encoding potassium ion transporters. Furthermore, MACPFs are restricted to certain species of *Pezizomycotina* and *Basidiomycota* and have multiple biological roles in cell adhesion and signaling (Humtsoe et al., 2005).

Based on our genome sequencing and gene annotation, we performed transcriptome analysis of *M. conica* SH in different development stages and mainly have three new discoveries: 1) RNA polymerase II TF activity was enriched in the mycelium specifically enriched gene cluster. As known, the initiation of pol II transcription is of particular interest because of the regulation of the process. Transcriptional activators and repressors exert their effects at this early stage in gene expression to influence cellular physiology and development (Liu et al., 2013). The enrichment of transcription system in the mycelium indicated that most genes might be active in the first stages and repressed in late stages rather than active in late stages. 2) The transition from vegetative mycelium to mature fruit body has undergone significant morphological changes. Genes encoding hydrophobins, CAZymes, and transcriptional factors were proven to be essential in this biological progress. In *Morchella*, genes involved in the vegetative growth of *M. importuna* have been investigated previously and DEGs have been found in primary metabolism processes, such as carbohydrates, fatty acids, proteins, and energy metabolism. Major developmental switch started in an earlier stage of fruit body growth, and less differences were found in later stages (Liu et al., 2019). KEGG analysis of genes significantly upregulated in the fruiting body suggested that they were mainly involved in other glycan degradation and ABC transporters. MFS and ABC transporters are usually involved in the transport of different substrates and important in defending pathogen (Morschhauser, 2010). The upregulation of these genes suggested that increasing disease resistance plays an important role in the development. 3) We also checked the genes annotated in our study and found that the downregulated protease gene was deubiquitinating enzyme Uch2 (*scaffold2.t334*) from C12 family, indicating an 850-fold reduction in fruit body. Many deubiquitinating enzymes are associated with the 26S proteasome contributing to the regulation of the activity of particle, and they are critical in the response to stresses such as nutrient starvation and heat shock (Komander et al., 2009). Attributed to the low-nutrition environment during the growth of fruit body, the deubiquitinating enzyme may regulate stress responses and the corresponding signal transductions. Among the lipases, Alpha esterase gene (*scaffold15.t73*) from abH01 family was significantly upregulated in fruit body, and the function of those epoxide hydrolases in fungi was unknown. They may play an important role in lipid metabolism during mycelium growth and providing *M. conica* SH the capability of degrading its host cell walls during colonization. On the other hand, kinase Akt from AGC family was enriched in *Morchella* species. Specifically, family plays a critical role in cell growth and survival (Kane and Weiss, 2003), and fungi employ PKA pathway in a variety of processes including control of differentiation and sexual development (Xu and Hamer, 1996). Furthermore, when comparing the TFs between *T. melanosporum* and *M. conica* SH, only three homologous TFs (TmelHMS1, TmelMBF1, and TmelHAA1) in *M. conica* SH showed significant upregulation in the fruiting body (fold change > 200). As known, TmelHMS1 functions as a regulator of cell morphology and TmelHaa1 as a weak acid stress response regulator. This is in contrast to what is observed in *T. melanosporum*: the TmeHMS1 was upregulated in ectomycorrhizas instead of fruit body (Montanini et al., 2011), indicating this homolog may be a critical and common regulator in different morphologies. Furthermore, the formation of the fruiting body is a highly complex developmental process, and the mating-type loci are the master regulators in fungi (Montanini et al., 2011). Homologs (Scaffold86.t1) of a fruit body regulator protein MAT-1-2-1 (Martin et al., 2010) are present in the *M. conica* SH genome; however, the expression levels of these genes are low in both the mycelium and fruit body.

Many studies have noted beneficial interactions between bacteria and other mushrooms; endophytes exist at a certain or the entire stage of the host and can facilitate the growth and enhance the stress resistance of the host. Previous research mainly focused on the bacterial communities in the soil; in this study, we firstly detect the endophytic bacterial communities in different development stages of the fruiting body in *M. conica* SH. Our results showed that *Flavobacterium*, *Pseudomonas*, and *Pedobacter* were dominant in the whole development stages of the fruiting bodies. The high abundance of these endophytic bacterial communities raises questions concerning their roles in the development of *M. conica* SH. It is reported that *Pedobacter*, *Pseudomonas*, *Stenotrophomonas*, and *Flavobacterium* were dominant in the microbiome of *M. sextelata* cultivated soil (Benucci et al., 2019), and *Pseudomonas*, *Flavobacterium*, *Janthinobacterium*, and *Polaromonas* were also detected in the fruiting bodies of Pezizales truffle species (Benucci and Bonito, 2016; Splivallo et al., 2019), including *Kalapuya brunnea*, which belongs to the Morchellaceae family (Trappe et al., 2010). The role of *Flavobacterium*, *Pseudomonas*, and *Pedobacter* in the development of indicated species indicated that they were conserved in the *Morchella*. Additionally, *Massilia* and *Herbaspirillum* showed distinct patterns in the first three stages and mature stages. As reported, *Massilia* was involved in the synthesis of multiple secondary metabolites and enzymes (Feng et al., 2016; Zheng et al., 2017), while *Herbaspirillum* was involved in nitrogen fixation (Hoseinzade et al., 2016). The obvious differences in abundance and functions of these two endophytic bacterial communities provide a direction for our future research. We will investigate the amount of different endophytic bacterial communities in the early and late stages and identification of the function of endophytic bacteria. Then, by cultivating endophytic bacteria with specific functions such as promoting growth, resisting pathogens, or producing active substances (Yuan et al., 2018; Ullah et al., 2019) by putting these bacteria back (injection or co-cultivation) into the host, they could complete their special function (Afzal et al., 2019; Eke et al., 2019).

Endophytic bacteria play many important roles during the whole life of the host, with biological effects on plant growth and development and disease resistance (Ryan et al., 2008). Endophytic bacteria promote plant growth and development by benefiting plants in getting nutrients, and the other is to modulate the production of growth and development-related hormones (Ma et al., 2016). Endophytic bacteria can protect plants from the attack of pathogens by inhibiting the growth of plant pathogens, such as the production of antibiotics and lysozymes (Miliute et al., 2015), causing the pathogens to lack nutrients and initiating plant defense mechanisms (Afzal et al., 2019).

Finally, in order to further verify the role of endophytic bacterial communities in the growth and development of *M. conica* SH, we analyzed the relationship between the abundance of endophytic bacterial communities and gene expression at different development stages of the fruiting body and found that several endophytic bacterial communities, including *Herbaspirillum*, *Pedobacte*r, *Arsenicitalea*, and *Duganella*, showed high correlation with modules enriched for “cell wall” and “proteasome regulatory subunit”; cell wall synthesis and remodeling are a common function during the fruiting body development (Krizsan et al., 2019). Proteasome is regarded as an important regulatory mechanism in cell cycle and growth and is highly expressed in several fruiting body-formatted fungi (Yamada et al., 2006; Wang et al., 2013). Taken together, our findings suggested that bacterial communities might promote the development of the fruiting body through regulating specific gene expressions in pathways relating to cell wall synthesis and proteasome of *M. conica* SH. Further studies are warranted for unraveling the potential role of endophytic bacteria in the fruiting body development.

## Conclusion

In this study, we first reported the genome and transcriptomes of the medicinal edible fungi *M. conica* SH and provide essential tools to unravel the mechanisms of substrate degradation and fruit body formation. Transcriptome analysis reveals significant differences between the mycelia and fruiting body. ABC transporters and metabolism-related genes are responsible for the mycelia and fruiting body, respectively. Besides, we first detected the endophytic bacterial communities in the fruiting body. Dynamic shifts of bacterial communities during different development stages of the fruiting body were detected, and co-expression analysis suggested that bacterial communities might play an important role in regulating specific gene expressions. In summary, our study provided a direction for artificial cultivation of *M. conica* SH on gene expression and bacterial communities.

## Data Availability Statement

The datasets presented in this study can be found in online repositories. The names of the repository/repositories and accession number(s) can be found in the article/Supplementary Material.

## Author Contributions

BL, GW, YS, LZ, and XiW analyzed the genomic data and were responsible for the experiment. PL, JW, HL, and AP analyzed the metagenomic information. YZ, XL, and WC provided the samples. XL and WC validated of gene expression by qRT-PCR. BL, GW, YS, and QM wrote the manuscript. LS and YH edited the manuscript. YY, CZ, and XuW analyzed the mitochondrial genome information of *M. conica* SH and added orthology analysis of *M. importuna* and *M. sextelata*. CZ and XuW analyzed the genome information and edited the revised manuscript. XT conceived the experimental design and edited the manuscript. All authors contributed to the article and approved the submitted version.

## Conflict of Interest

The authors declare that the research was conducted in the absence of any commercial or financial relationships that could be construed as a potential conflict of interest.

## Footnotes

1 http://www.repeatmasker.org/

2 https://www.girinst.org/

3 http://www.cbs.dtu.dk/services/SignalP/

4 http://www.tcdb.org

5 http://kinase.com/

6 http://ftfd.snu.ac.kr

7 https://mycoclap.fungalgenomics.ca

8 http://github.com/lh3/seqtk

9 http://merops.sanger.ac.uk
