# Supplementary material for: Comparative Transcriptome and Endophytic Bacterial Community Analysis of Morchella conica SH
Source: Front Microbiol. 2021 Jul 20;12:682356. doi: 10.3389/fmicb.2021.682356 (PMC8329594; doi:10.3389/fmicb.2021.682356)

Supplementary information

Supplementary Table S1. Construction of libraries, generation and filtering of sequencing data used for genome assembly

Insert size (bp)	Read length (bp)	Raw data (Gb)	Filtered data (Gb)	Coverage (×)
**400**	250	5.06	3.78	67.54
**700**	250	1.06	0.86	15.36
**2k**	200	0.43	0.40	7.14
**5k**	230	0.95	0.89	15.89
**8k**	230	0.48	0.45	8.04
**Total**	-	7.98	6.38	113.97

Supplementary Table S2. Statistics of repeat contents in the *M. conica* SH genome

Type	Length (bp)	Percent (%)
DNA transposon	118,747	0.23
LINE	79,012	0.15
SINE	325	0.01
LTR	459,788	0.89
Satellite	8,588	0.02
Simple-repeat	36,239	0.07
Unclassified element	61,999	0.12
Total	764,725	1.49

Supplementary Table S3. Estimation of *M. conica* SH genome size based on K-mer statistics

K*	K-mer number	Peak depth	Genome size (bp)	Average read length	Total read number
**17**	196,780,041	74	55,967,964	210.29	22,051,316

*The frequency distribution of 17-mers within the raw read sequences displays 2 major peaks (A and B). Peak A, resembles a Gaussian distribution and represents k-mers of ~0-10X coverage which arise by chance due to sequencing errors. Peak B, corresponding to k-mers of ~20-5-X coverage, represents the majority of the genome and resembles a Poisson distribution with monor differences due to sequencing errors, heterozygosity and repetitive DNA. The total genome size of *M. conica* was estimated by obtaining the multiplication product of 17bp and the k-mer frequency (value at y-axis) corresponding to the coverage (value at x-asix) at Peak B.

Supplementary Table S4. Summary for annotation of predicted protein-coding genes in the *M. conica* SH genome assembly

	Number	Percent (%)
Total	9,676	100.0
NR	7,713	79.7
KEGG	5,241	54.2
KOG	5,611	58.0
GO	3,042	31.4
Annotated	7,713	79.7
Unannotated	1,963	20.3

Supplementary Table S24 The primers used in qRT-PCR

**Gene ID**	**Primer** Primer-F	Primer-R
scaffold23.t89	CATCCTGGCCTGTGTTCCAT	CGCCGCTCGATCCAAAT
scaffold30.T27	TCGTTGACGCGCAAGGT	GCACTACCAAAGCCGGCATA
scaffold5.t30	CTTCTGATGCTCTTATCCGTGATC	GTGCGCGGAAGAACACAAC
scaffold20.t83	CATTGTCATCTGGCATACATTCG	GCGGGCATAACAGGGAAGT
scaffold40.t14	CCCGCCATCGCAATTG	CCGCCGGAGAGGGTAAA
scaffold35.t11	AAGCTTGCAGTCGCCCAAT	TTTTCAGGAGCTCCATGGTCTT
scaffold3.t87(CYC3)	CCCAGAACCGCCGAGAA	TGGATCCGGCATAACCGTAT

All the following tables were upload as an excel spread sheet

Supplemental Table S5. List of single-copy orthology genes.

Supplemental Table S6. List of the fungi utilized in this study

Supplemental Table S7. Proteases in different fungal genomes, arranged by MEROPS family

Supplemental Table S8. Secreted proteases in different fungal genomes, arranged by MEROPS family

Supplemental Table S9. Lipases in different fungal genomes, arranged by lipase family

Supplemental Table S10. Secreted lipases in different fungal genomes, arranged by lipase family

Supplemental Table S11. Carbohydrate-degrading enzymes (CAZyme) in different fungal genomes, arranged by GH family

Supplemental Table S12. Secreted carbohydrate-degrading enzymes (CAZyme) in different fungal genomes, arranged by GH family

Supplemental Table S13. Lignocellulose-active proteins in Pezizomycetes and other fungal genomes, arranged by CAZyme family

Supplemental Table S14. Secondary metabolism clusters in different fungal genomes

Supplemental Table S15. Transporters in Pezizomycetes and other fungal genomes, predicted by TransportDB, arranged by function

Supplemental Table S16. All predicted transporters in Pezizomycetes and other fungal genomes, arranged by family

Supplemental Table S17. Kinases in Pezizomycetes and other fungal genomes, arranged by function

Supplemental Table S18. Transcription factors in Pezizomycetes and other fungal genomes, arranged by FTFD family

Supplemental Table S19. Putative transcription factors in *M. conica* SH vs *T. melanosporum*

Supplemental Table S20. Genome-wide expression analysis of *M. conica* SH genes measured in five development stages using RNA-Seq

Supplemental Table S21. K-means cluster of *M. conica* SH differential expression genes measured in five development stages and their GO function

Supplemental Table S22 Top 50 of the most up-regulated genes from *M. conica* SH mycelium stage (X0) comparing to fruit body stages (X1-X4)

Supplemental Table S23. Top 50 of the most down-regulated genes from *M. conica* SH mycelium stage (X0) comparing to fruit body stages (X1-X4)

Supplemental Table S25. The bacterial communities in fruiting bodies of *M. conica* SH at different developmental stages

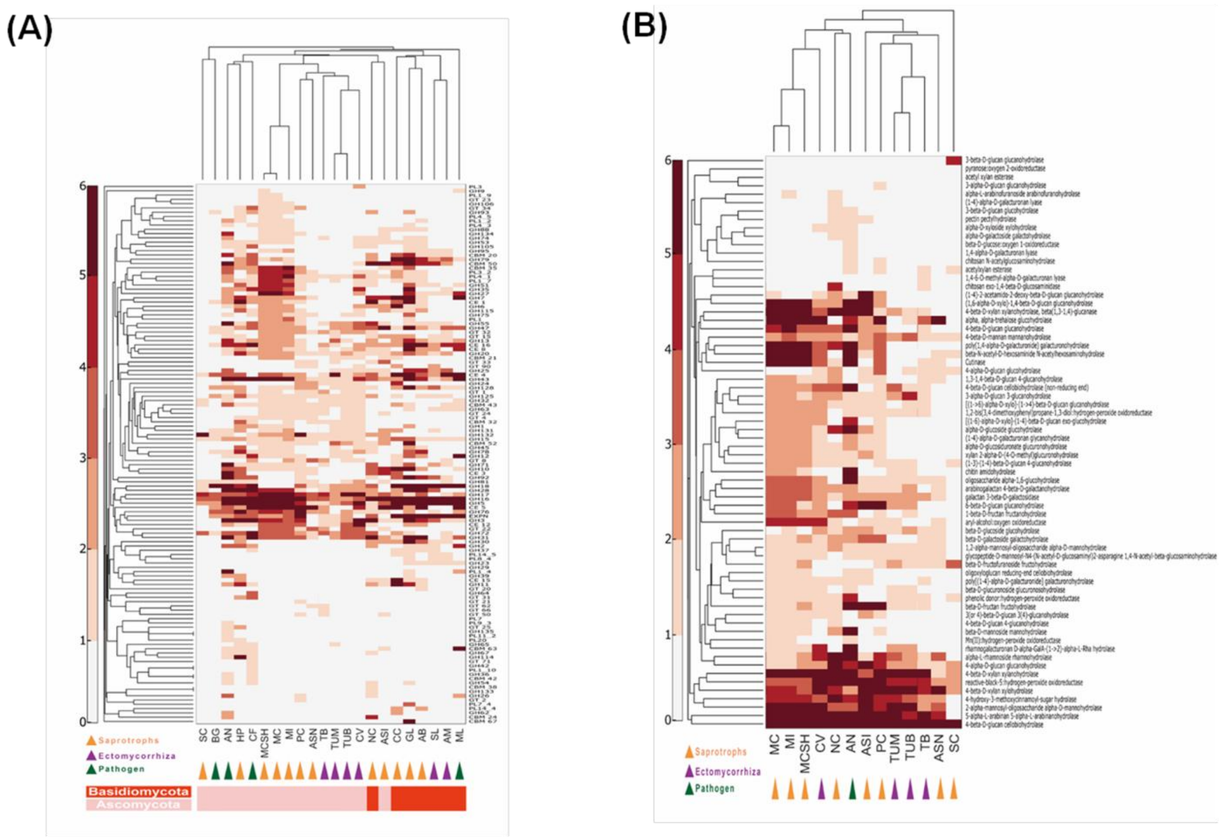

Supplemental Figure S1. Double hierarchical clustering of genes in representative fungal genomes. A double hierarchical clustering of the number of **(A)** secreted Carbohydrate-Active Enzymes (CAZyme) coding gene **(B)** cell wall degrading enzymes coding genes for each of the fungal species was performed using the MatLab software. The Euclidian distance between gene counts was used as distance metric and a complete linkage clustering was performed. The relative abundance of genes is represented by a color scale (on the left), from the minimum (white) to the maximum (red) nunmber of copies per species. Table S1 shows the full names of pecies and lifestyles.

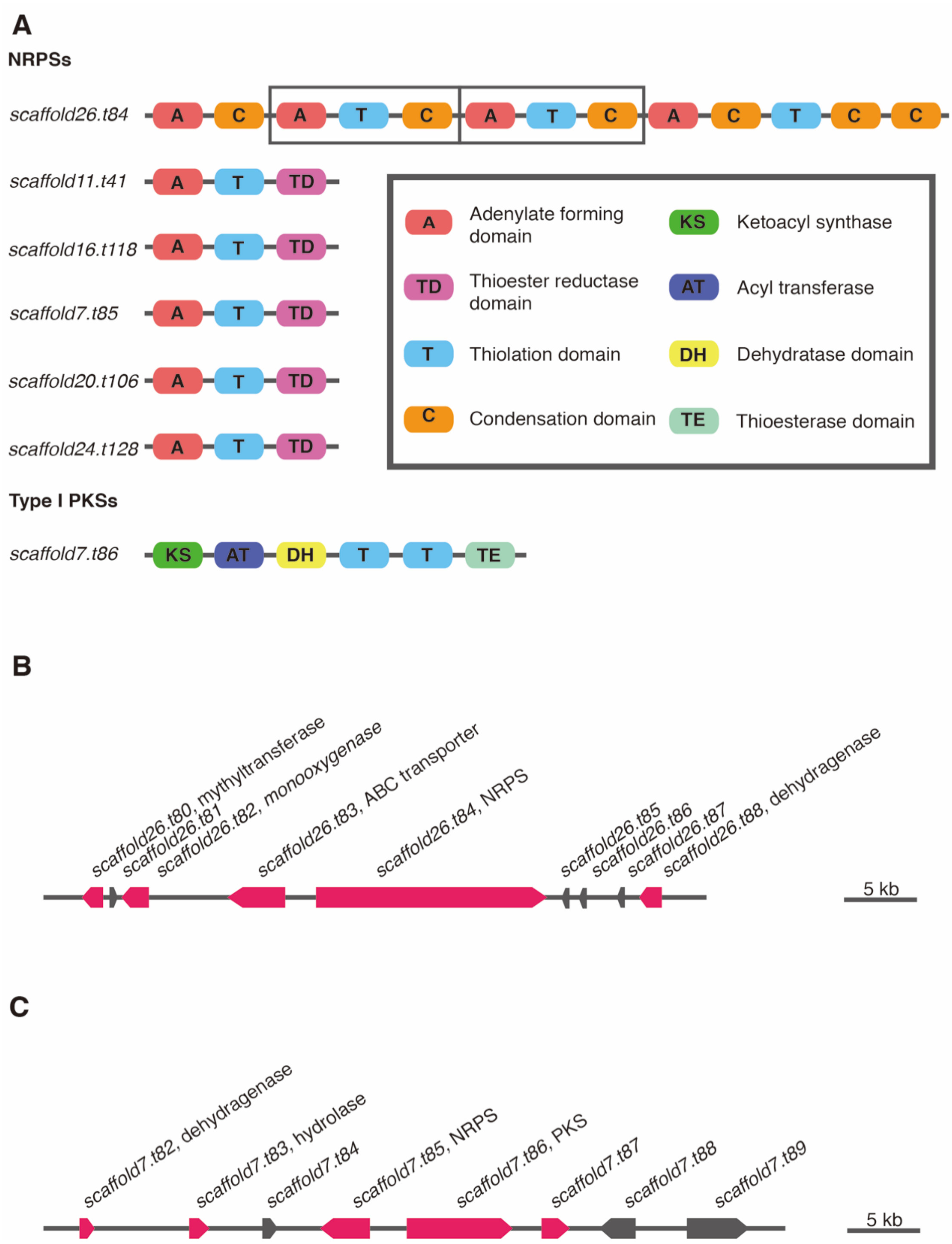

Supplemental Figure S2. Genes for polyketide and non-ribosomal peptide biosynthesis in *M. conica* SH. (**A**) Protein domain organization of all predicted PKSs or NRPSs. The putative siderophore biosynthesis scaffold26.t84 has two typical domain structures (A-T-C module are boxed), and the rest five NRPSs have domain structures A-T-TD module. (**B**) Putative biosynthetic gene cluster containing the NRPS gene *scaffold26.t84*. Genes with putative functions in siderophore biosynthesis are shown in pink. (**C**) Putative biosynthetic gene cluster containing the NRPS gene *scaffold7.t85* and the PKS gene *scaffold7.t86*. Genes with putative functions are shown in pink.

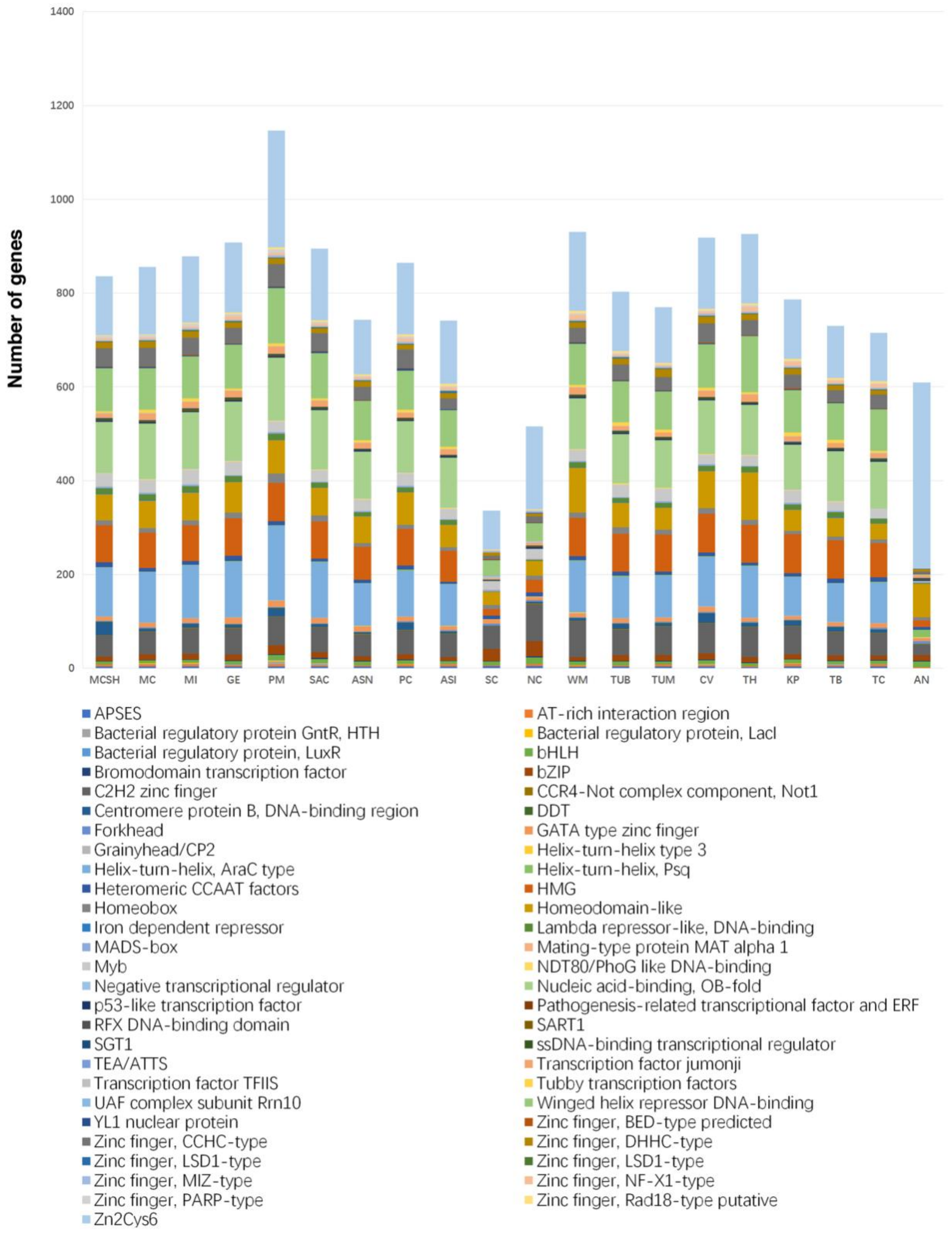

Supplemental Figure S3. Distribution of *Morchella conica* SH transcription factors (TFs) among different structural categories and comparison with the TF repertoire of pezizomycetes and other fungi.

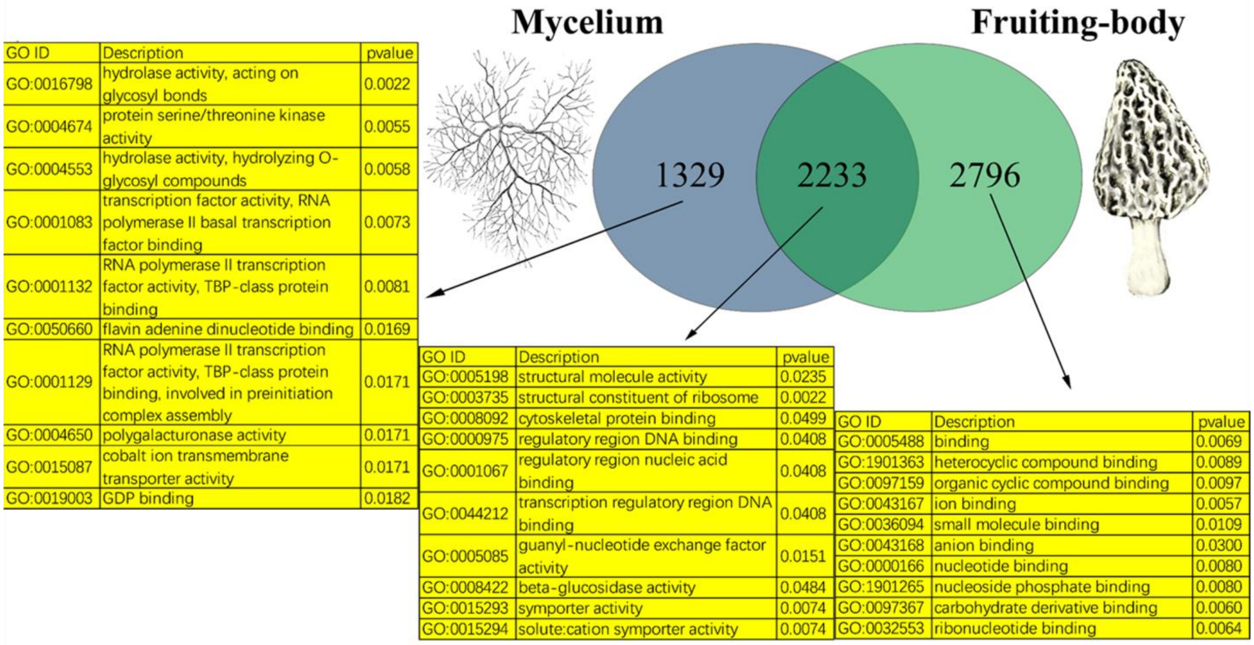

GO ID	Description	pvalue
GO:0016798	hydrolase activity, acting on glycosyl bonds	0.0022
GO:0004674	protein serine/threonine kinase activity	0.0055
GO:0004553	hydrolase activity, hydrolyzing O-glycosyl compounds	0.0058
GO:0001083	transcription factor activity, RNA polymerase II basal transcription factor binding	0.0073
GO:0001132	RNA polymerase II transcription factor activity, TBP-class protein binding	0.0081
GO:0050660	flavin adenine dinucleotide binding	0.0169
GO:0001129	RNA polymerase II transcription factor activity, TBP-class protein binding, involved in preinitiation complex assembly	0.0171
GO:0004650	polygalacturonase activity	0.0171
GO:0015087	cobalt ion transmembrane transporter activity	0.0171
GO:0019003	GDP binding	0.0182

GO ID	Description	pvalue
GO:0005198	structural molecule activity	0.0235
GO:0003735	structural constituent of ribosome	0.0022
GO:0008092	cytoskeletal protein binding	0.0499
GO:0000975	regulatory region DNA binding	0.0408
GO:0001067	regulatory region nucleic acid binding	0.0408
GO:0044212	transcription regulatory region DNA binding	0.0408
GO:0005085	guanyl-nucleotide exchange factor activity	0.0151
GO:0008422	beta-glucosidase activity	0.0484
GO:0015293	symporter activity	0.0074
GO:0015294	solute:cation symporter activity	0.0074

GO ID	Description	pvalue
GO:0005488	binding	0.0069
GO:1901363	heterocyclic compound binding	0.0089
GO:0097159	organic cyclic compound binding	0.0097
GO:0043167	ion binding	0.0057
GO:0036094	small molecule binding	0.0109
GO:0043168	anion binding	0.0300
GO:0000166	nucleotide binding	0.0080
GO:1901265	nucleoside phosphate binding	0.0080
GO:0097367	carbohydrate derivative binding	0.0060
GO:0032553	ribonucleotide binding	0.0064

Supplemental Figure S4. GO enrichment for differential expressed genes groups.

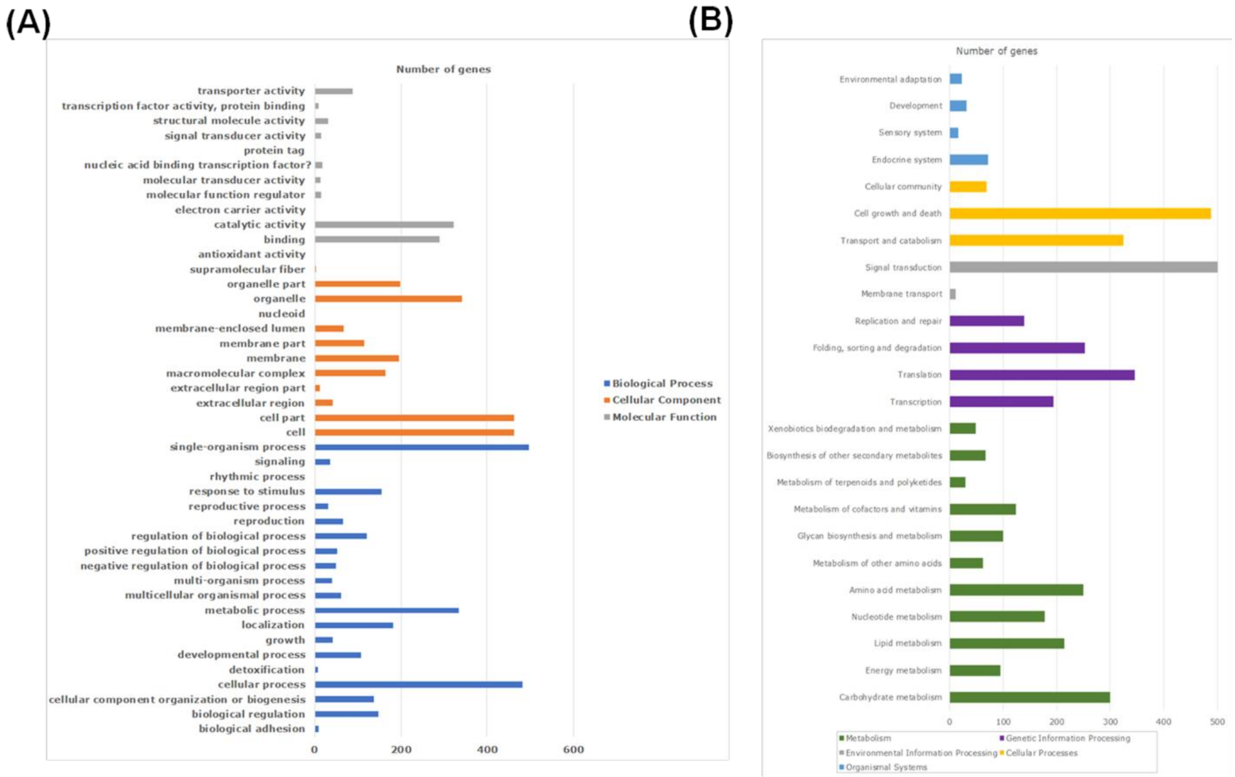

Supplemental Figure S5. Classification of the *M. conica* SH transcriptome. (A) Histogram of the GO annotation. Genes were grouped into three major GO categories, which were indicated by different colored columns. Y-axis indicates the number of unigenes in a category. **(B)** Histogram of the KEGG annotation see. Genes were grouped into five major KEGG pathway categories, which were indicated by different colored columns. Y-axis indicates the number of unigenes in a category.

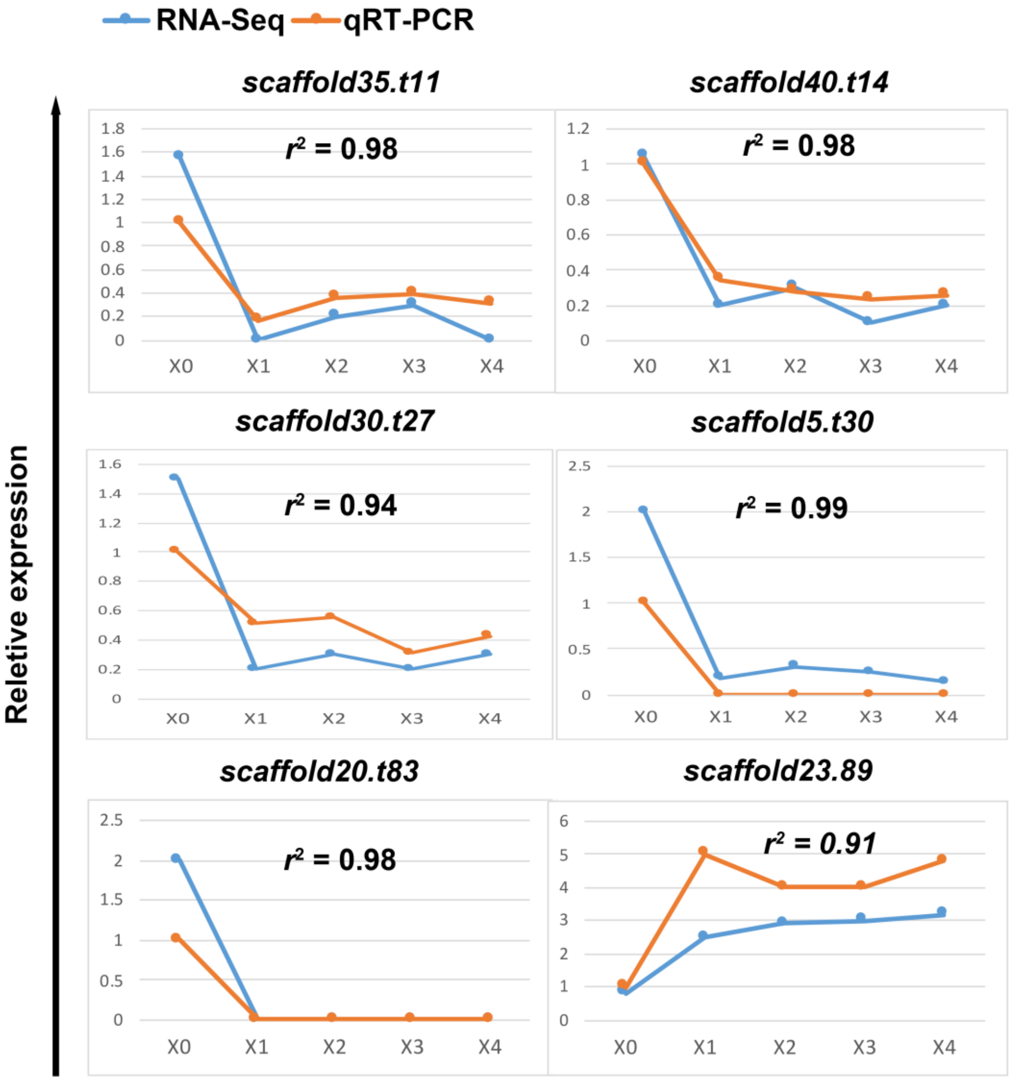

Supplementary Figure S6. Quantitative real-time PCR (qRT-PCR) validation of the expression of differentially expressed genes identified using RNA-sequencing..

Supplementary Result 1

Gene Content of the Mitochondrial DNA in *M. conica* SH

The mitochondrial genome of *M. conica* SH was extracted from whole genome sequence, the final assembly resulted in a scaffold of 262,110 bp (scaffold68), representing a circular molecule with a GC content of 38.67%. Which shares significant similarity with mitochondrial genome from *M. importuna* SCYDJ1-A1 (available through the *M. importuna* genome portal at the JGI MycoCosm database, JGI project ID: 1047733) (**Figure 1**). However, only partial amino acids sequences were aligned to reported mitochondrial gene contents by BLASTX (**Table 1**).

Table 1. Conserved protein-coding genes in *M. conica* SH mitochondrial genome.

Gene	Position	*E*-value	Identity	Similarity	Accession
atp6	36,008-36,757	5e-131	198/250 (79%)	*Phialocephala subalpina*	YP_004733041.1
cob	60,035-60,337	1e-10	91/101 (90%)	*Cercospora beticola*	AFN43049.1
cox1	132,067-132,366	2e-52	87/100 (87%)	*Phialocephala helvetica*	AET27895.1
	115,487-115,810	3e-60	103/107 (96%)	*Mycocalicium subtile*	ASW34448.1
cox2	179,211-179,567	3e-74	106/119 (89%)	*Pyronema omphalodes*	YP_009240567.1
cox3	170,738-170,974	6e-42	73/79 (92%)	*Pyronema omphalodes*	YP_009240565.1
matR	74,443-74,877	4e-60	93/145 (64%)	*Ophiocordyceps sinensis*	YP_009364296.1
	41,610-42,041	2e-71	112/144 (78%)	*Venturia inaequalis*	AAB95256.1
	99,618-100,025	1e-55	87/135 (64%)	*Magnusiomyces ingens*	YP_009029663.1
nad1	17,638-17,973	1e-59	94/105 (90%)	*Microbacterium laevaniformans*	WP_005050865.1
	25,649-25,906	9e-41	67/82 (82%)	*Marssonina brunnea*	YP_004842022.1
	30,744-31,118	9e-50	84/125 (67%)	*Pyronema omphalodes*	YP_009240530.1
nad2	76,274-77,167	4e-120	201/306 (82%)	*Phialocephala subalpina*	YP_004733047.1
nad4	86,930-87,880	4e-169	253/314 (81%)	*Pyronema omphalodes*	YP_00924053.1
nad5	201,666-202,505	1e-176	254/281 (90%)	*Tuber melanosporum*	XP_002839371.1
nad6	161,152-161,673	9e-52	99/180 (55%)	*Mycocalicium subtile*	ASW34455.1

rps5	2,607-3,056	5e-21	49/90 (54%)	*Talaromyces marneffei*	NP_943722.1

Figure 1. Dot plot showing a synteny comparison between mitochondrial DNAs of *M. conica* SH (yangmt ffold 68) and *M. importuna* (MIMmt ffold_1).

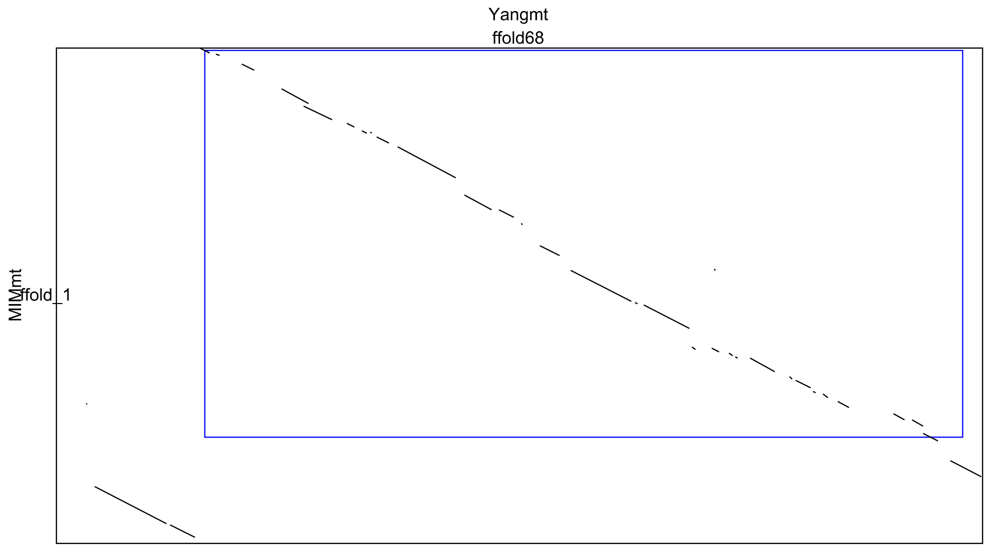

Supplement: Supplementary file 1 [file Data_Sheet_1.PDF]
